# Identification and Validation of Immune-Related Gene Prognostic Signature for Hepatocellular Carcinoma

**DOI:** 10.1155/2020/5494858

**Published:** 2020-03-07

**Authors:** Wenbiao Chen, Minglin Ou, Donge Tang, Yong Dai, Weibo Du

**Affiliations:** ^1^State Key Laboratory for Diagnosis and Treatment of Infectious Diseases, National Clinical Research Center for Infectious Disease, Collaborative Innovation Center for Diagnosis and Treatment of Infectious Diseases, The First Affiliated Hospital, School of Medicine, Zhejiang University, Hangzhou 310003, China; ^2^Clinical Medical Research Center of The Second Clinical Medical College, Jinan University, Shenzhen People's Hospital, No. 1017, Dongmen North Road, Luohu District, Shenzhen 518020, China; ^3^Scientific Research Center, The Second Affiliated Hospital of Guilin Medical University, Guilin 541199, China

## Abstract

Immune-related genes (IRGs) have been identified as critical drivers of the initiation and progression of hepatocellular carcinoma (HCC). This study is aimed at constructing an IRG signature for HCC and validating its prognostic value in clinical application. The prognostic signature was developed by integrating multiple IRG expression data sets from TCGA and GEO databases. The IRGs were then combined with clinical features to validate the robustness of the prognostic signature through bioinformatics tools. A total of 1039 IRGs were identified in the 657 HCC samples. Subsequently, the IRGs were subjected to univariate Cox regression and LASSO Cox regression analyses in the training set to construct an IRG signature comprising nine immune-related gene pairs (IRGPs). Functional analyses revealed that the nine IRGPs were associated with tumor immune mechanisms, including cell proliferation, cell-mediated immunity, and tumorigenesis signal pathway. Concerning the overall survival rate, the IRGPs distinctly grouped the HCC samples into the high- and low-risk groups. Also, we found that the risk score based on nine IRGPs was related to clinical and pathologic factors and remained a valid independent prognostic signature after adjusting for tumor TNM, grade, and grade in multivariate Cox regression analyses. The prognostic value of the nine IRGPs was further validated by forest and nomogram plots, which revealed that it was superior to the tumor TNM, grade, and stage. Our findings suggest that the nine-IRGP signature can be effective in determining the disease outcomes of HCC patients.

## 1. Introduction

Hepatocellular carcinoma (HCC) is a common cancer of the liver and one of the leading causes of cancer-associated mortality worldwide [1]. Currently, surgical resection is the primary treatment option for the condition. However, because of late diagnosis, the postoperative survival rate of patients is still low and the recurrence rate is remains high. Given the lack of specific symptoms in the early stage of the disease, patients are often diagnosed when the disease has advanced to middle and late stages. This leads to a low 5-year survival rate of 40%~50% if patients do not receive radical treatment. On the contrary, HCC patients who are diagnosed early have a relatively good prognosis with a 5-year survival rate of about 90% after surgery [2, 3]. However, the traditional diagnostic biomarkers of HCC are limited in sensitivity and specificity. And this has prevented early diagnosis and treatment of this disease [4]. Therefore, it is urgent to find a novel clinical signature that is closely associated with the occurrence and development of HCC for better prediction of the recurrence, metastasis, and prognosis of patients. This will ensure early diagnosis timely and treatment of the condition.

Previously, the clinical survival stratification of HCC patients was based on features comprising molecular markers, such as gene, miRNA, and lncRNA. Cai et al. reported a negative correlation between the expression levels of RAD21, CDK1, and HDAC2 and the survival time of HCC patients [5]. Also, six lncRNAs that can predict the survival rate of HCC patients by grouping them into high- or low-risk groups have been suggested [6]. These molecular markers are not only useful in tracking the prognosis of HCC patients but also crucial complements for the clinical and pathological staging of tumors [7, 8]. However, given that this concept was based on a relatively small data set and was short of sufficient validation, it has not been adopted in clinical practice [9]. The emergence of publicly available resource-sharing gene expression databases has provided a platform to investigating more reliable biomarkers of HCC biomarkers. However, data mined from these databases may not be accurate because of the high biological heterogeneity, gene expression differences, and technical biases between the databases and measurement platforms [10]. To transcend this challenge, bioinformatics tools based on big data together with multigroup analysis have enabled effective data preprocessing and mining for the identification of prognostic tumor markers [11].

Recent studies have shown that the immune system, including immune cells, immune factors, and immune microenvironment, are essential factors in tumorigenesis [12]. Besides, tumor-related immunity exists in all stages of tumorigenesis. And its effects include destroying genome stability, apparent genetic modification, promoting the proliferation of tumor cells, resisting tumor anti-apoptosis, stimulating angiogenesis, and shaping tumor micro-growth environment [13]. Hepatocellular carcinoma can be initiated by infectious diseases, especially chronic inflammation caused by the hepatitis virus can induce fibrosis or cirrhosis and subsequent tumorigenesis [14]. The liver is a vital immune organ and is therefore rich in various natural immune cells, which play an essential role in the maintenance of normal immune function of human body. Under normal conditions, the liver's immune system can recognize pathogens and remove tumor cells from the tumor microenvironment. However, under pathological conditions, HCC cells can suppress the immune system leading to the proliferation of tumor cells and immune deficiency [14, 15]. Studies on genomics regarding the immune mechanism of HCC have led to the identification of molecular markers that can predict immune checkpoint blockade reactivity. Further studies on these biomarkers are ongoing and may improve the accuracy of immunotherapy [15]. At present, immunotherapies such as programmed-death 1 (PD-1) and programmed death-ligand 1 (PDL-1) are showing great success in the clinical treatment of HCC [16].

Given the role of immune mechanisms in the pathogenesis of HCC, studies based on immune genes, immune microenvironment, immune infiltrating cell composition, immune checkpoint, and immunotherapeutic targets have been applied and conducted in clinical trials. A study by Sia et al. on the immune-specific class of HCC divided HCC into two distinct groups based on immune-related genomic signals. The HCC patients who were in the immune class showed a high degree of immunohistochemical expression of PD-1/PD-L1. However, the two distinct immune groups showed different components of the tumor microenvironment and exhibited active and exhausted immune response, which might represent the ideal candidates to receive immunotherapy [17]. Furthermore, by combining molecular and histological analysis of HCC, Calderaro and his colleagues made a more detailed classification of HCC based on molecular subgroups, histological features, genetic alterations, and oncogenic pathways. They noted that the immune classification by Sia et al. corresponded to different pathological stages, immune cell infiltration, gene mutation, and tumor pathway related to the different prognosis of HCC patients [18]. Although the HCC classifications based on molecular data are now well established, the knowledge of molecular features has not yet been applied in the identification of HCC biomarkers and requires further research. In this study, by combining the multigene expression data sets, we established a nine-IRGP signature to predict the individual prognostic characteristics of HCC. For validation of the signature, we investigated its accuracy and efficiency in determining the prognosis of HCC patients in combination with clinical features. The findings of this study showed and proved that the nine-IRGP signature can be applied in the clinical prognosis of HCC patients.

## 2. Materials and Methods

### 2.1. Data Mining and Processing

Three public data sets of HCC, one from The Cancer Genome Atlas (TCGA) RNA-seq and two Gene Expression Omnibus (GEO) data sets (GSE14520, GSE76427), containing genes expression profile and clinical follow-up information were used in this retrospective study. Factors that could have influenced the results of the study, such as radiation therapy, targeted drug therapy, chemical drug interventional therapy, and immunotherapy, were excluded. The preprocessing of TCGA RNA-seq was as follows: (1) HCC samples without clinical information or in which the overall survival (OS) was zero were removed; (2) data on normal HCC tissue samples were removed; (3) the genes in which the Fragments per Kilobase Million (FPKM) were zero in more than half of the HCC samples were excluded. The GEO data were preprocessed as follows: (1) data on normal HCC tissue samples were removed, whereas data on primary tumor were retained; (2) the OS period was converted from year/month to day; (3) mapping microarray probe to human gene SYMBOL by bioconductor package; (4) and only the expression profile of immune-related genes was included. The GSE14520 and GSE76427 data sets were merged into an independent verification data set. The TCGA data set was randomly divided into the training and testing sets based on the following conditions: (1) All TCGA samples were randomly divided 100 times in advance. The training set samples were analyzed as follows: testing test set = 0.5 : 0.5 ratio. (2) The distribution of age, clinical stage, follow-up time, and death rate was similar between the two data sets. (3) After clustering of gene expression profiles of two random data sets, the number of HCC samples in the dichotomies was similar. This study was approved by the Clinical Research Ethics Committee of Shenzhen People's Hospital.

### 2.2. Construction of the IRGP Prognostic Signature

A collection of immune-related genes were downloaded from the InnateDB database (http://www.innatedb.com/) then subjected to manual correction. These genes encoded proteins related to the endogenous immunity of several species reported in the literature. The endogenous immune-related genes of *human beings* which are involved in many immune processes, including cellular response to cytokine, cell mediation of immunity, immune signaling pathway, and immune response to tumor cells, were identified. The gene expression levels were compared pairwise in a particular sample or sequence to produce a score for each immune-related gene pair (IRGP). An IRGP was calculated as follows: (1) if IRG 1 < IRG 2, IRGP = 1; (2) otherwise, IRGP = 0. The advantage of analyzing genes in a pairwise approach is that it eliminates the need for standardization steps for individualized analysis. Some IRGPs were removed because they had a unique value of 0 or 1 among all samples in the data set to avoid biases and unrepeatability of the study.

The univariate Cox regression analysis model was used for each IRGPS, and survival data were analyzed using the R package survival coxph function. Next, univariate Cox regression analysis model was performed for clinical characters in the training set to identify IRGPs related to risk signature with log‐rank < 0.05. After univariate Cox regression analysis, we obtained many of IRGPs that were not suitable for clinical application. Therefore, the range of IRGPs was further reduced while maintaining high accuracy. Least absolute shrinkage and selection operator (LASSO) is a biased estimation tool for data with complex collinearity. It can select variables and estimate parameter simultaneously and better solve the multicollinearity problem in regression analysis [19]. Thus, we used the LASSO Cox regression analysis to decrease the number of IRGPs by R package glmnet.

### 2.3. Validation of the IRGP Prognostic Signature

The risk score for each HCC sample was calculated based on the IRGP prognostic signature using the following formula: risk score = expression_gene 1_ × *β*_gene 1_ + expression_gene 2_ × *β*_gene 2_+⋯+expression_gene *x*_ × *β*_gene *x*_, in which *x* was the number of IRGPs and *β* was coefficient value for each IRGPs. Taking the median value of risk score as the threshold, we divided all the HCC samples into high-risk or low-risk groups. The accuracy and sensitivity of survival prediction based on the risk score were verified by receiver operating characteristic (ROC) curve analysis and determined by the value of area under the curve (AUC) in 1, 3, and 5 years. Kaplan–Meier (KM) survival curves analysis (*p* < 0.01) was used to analyze the over survival (OS) of the high-risk and low-risk groups. We then integrated IRGPs with existing clinical and pathologic features for multivariate Cox regression analysis. Tumor TNM, stage, grade, age, and body mass index (BMI) were regarded as continuous variables. The association between IRGPs risk score, clinical, and pathologic features was determined by KM analysis. Prognostic risk models of tumor TNM, grade, age, and stage were constructed. Subsequently, a Cox proportional hazards regression model was constructed by combined the Tumor TNM, grade, age, stage, and risk scored. The R package rms was used to compare these models with the IRGP prognostic signature. Concordance index (C-index) was used to assess the accuracy of the prognostic biomarkers. Also, the comparison between IRGPs and clinical/pathologic features was performed by forest and nomogram plots to determine the effectiveness of the prognostic value. The statistical difference of IRGPs in the clinical/pathologic features was compared using the Kruskal–Wallis test. The functional roles of IRGPs were determined by Gene Ontology (GO) and Kyoto Encyclopedia of Genes and Genomes (KEGG) analysis using R package clusterprofiler.

## 3. Results

### 3.1. Construction of the IRGP Prognostic Signature

After raw data preprocessing, a total of 342, 220, and 95 HCC samples were retrieved from TCGA RNA-seq, GSE14520, and GSE76427 data set, respectively (Supplementary [Supplementary-material supplementary-material-1]). The TCGA RNA-seq was separated into the training set with 170 HCC samples and the testing set with 172 HCC samples (Supplementary [Supplementary-material supplementary-material-1]). A total of 38422 IRGPs were obtained from 1039 IRGs retrieved from the InnateDB database by gene pairwise calculation in the training set. For evaluating the difference in gene expression between TCGA RNA-seq and GEO database, the IRG and IRGP data were used to conduct cluster analysis of HCC samples from TCGA and GEO, respectively. According to the results, both IRGs and IRGPs significantly separated the data from the two platforms (Supplementary [Supplementary-material supplementary-material-1]A, B). Notably, the difference between TCGA RNA-seq and the GEO database was narrowed after conversion from IRG to the IRGP platform (Supplementary [Supplementary-material supplementary-material-1]C, D). IRGPs were able to distinguish gene expression differences. Moreover, the IRGPs which were calculated based on IRGs gene pairwise could effectively reduce the differences between databases.

**Figure 1:**
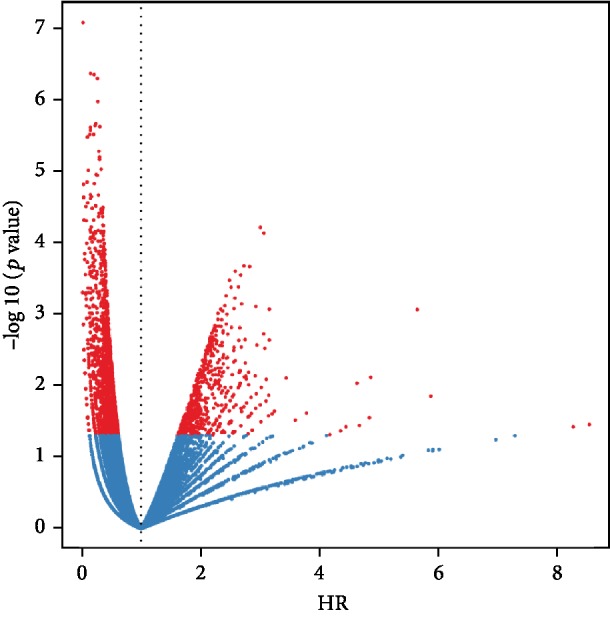
The association between *p* value and hazard ratio (HR) of 2716 IRGPS. Red nodes indicate IRGPs with log-rank *p* < 0.05.

Univariate Cox regression analysis was performed for the 38422 IRGPs, of which 2716 IRGPs showed significant prognostic potential (*p* < 0.05). We further analyzed the relationship between *p* value and hazard ratio (HR) and observed that HR corresponded to IRGPs with significant *p* value deviated from 1, indicating the prognostic value of the 2716 IRGPs (Figure 1). After that, we performed the LASSO Cox regression analysis to reduce the number of IRGPs in the risk model and finally obtained nine IRGPs for further study (Table 1).

### 3.2. Validation of the IRGP Prognostic Signature

**Figure 2:**
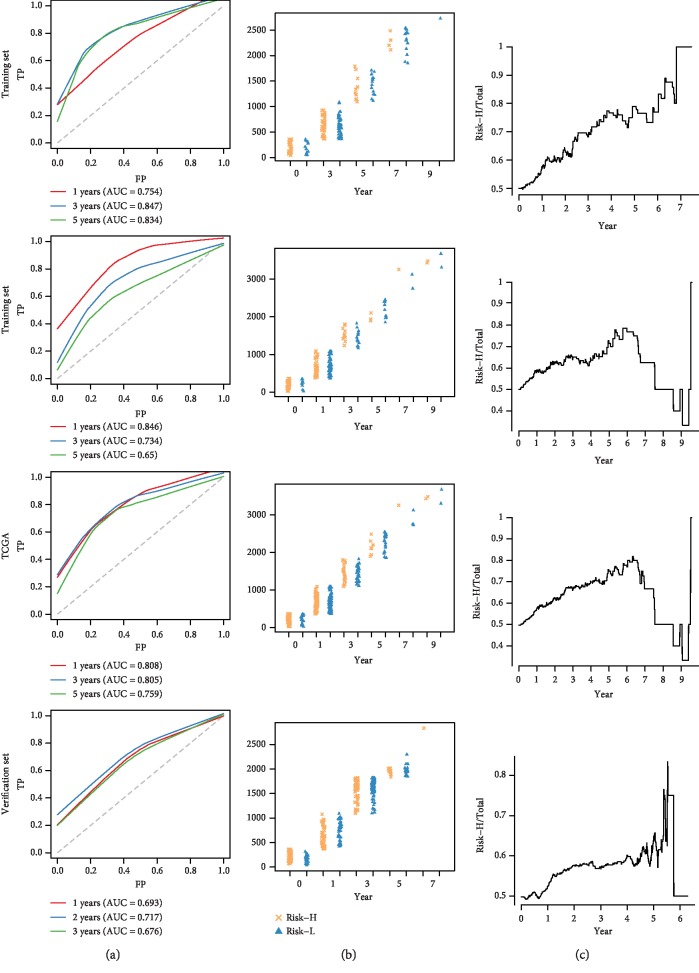
Prognostic analysis of the nine-IRGP signature. (a) Time-dependent ROC curve analysis of the nine-IRGP signature based on training, testing, TCGA, and verification sets. (b) Statistics of high- and low-risk groups under different over survival based on the training, testing, TCGA, and verification sets. (c) The proportion of the high-risk group in the total samples changed with over survival time in the training, testing, TCGA, and verification sets.

**Figure 3:**
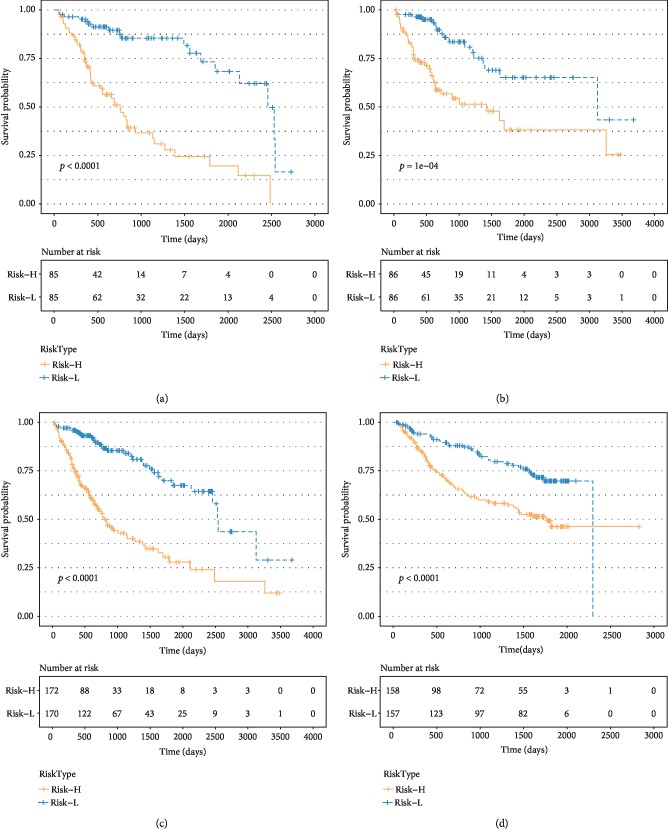
Kaplan–Meier survival analysis of the HCC samples based on the nine-IRGP signature. Kaplan–Meier curves show the survival time of the (a) training, (b) testing, (c) TCGA, and (d) verification sets.

We established ROC risk models based on the nine-IRGP signature for 1, 3, and 5 years. The mean value AUC of the training, testing, TCGA, and verification sets was 0.812, 0.743, 0.791, and 0.695, respectively (Figure 2(a)). The nine IRGPs grouped HCC patients into high- and low-risk groups based on OS. The distribution of HCC samples in the high- and low-risk groups was calculated under overall survival. No significant difference was observed in the sample size between the high- and low-risk groups at 0, 1, and 3 years. On the contrary, the HCC samples in the high-risk group were fewer than those in the low-risk group after the 5^th^ year (Figure 2(b)). Besides, with the prolongation of OS, the proportion of HCC samples in the high-risk group decreased gradually in relation to the total samples (Figure 2(c)). These findings were consistent with the clinical findings that HCC patients did not relapse within 5 years after therapy, the OS was greatly improved and the recurrence rate gradually reduced [20]. Also, we analyzed the OS of HCC samples based on the nine-IRGP signature using the KM curve. The *p* value of the training, testing, TCGA, and verification sets was <0.0001, 1*e*-04, <0.0001, and <0.0001, respectively, which indicated that there were significant differences in OS between the high and low groups in all data sets. The nine-IRGP signature was verified as useful prognostic tool as it could stratify HCC into the high- and low-risk groups. The OS of the high-risk group was shorter than the low-risk groups in all the data sets (Figure 3).

### 3.3. Association Analysis between the Nine IRGPs and Clinical Features

**Figure 4:**
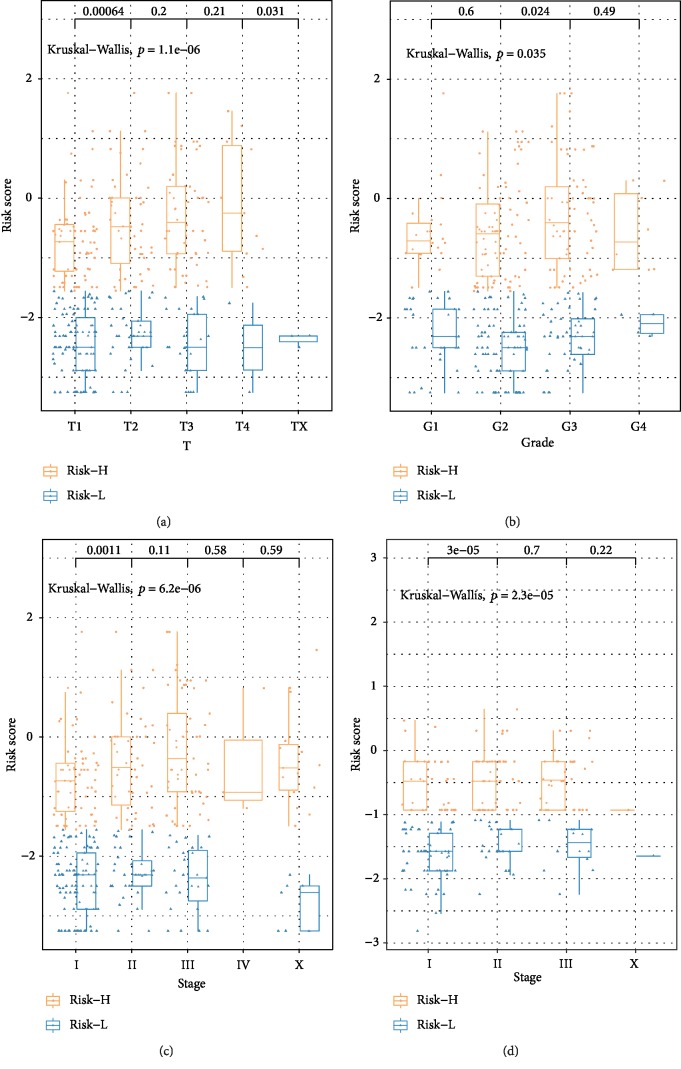
Association between clinical features (T, stage, and grade) and risk score based on the nine-IRGP signature. Distribution of risk scores in (a) T, (b) grade, and (c) stage for TCGA data set. (d) Distribution of risk scores in stage for GSE14520 data set.

The relationship between clinical features such as T, N, M, age, grade, BMI, stage and risk score was analyzed to confirm the accuracy of the nine-IRGP signature further. The distribution of risk score of T, grade, and stage in the TCGA database and stage in the GSE14520 showed that high-risk groups had a significantly higher risk score than low-risk groups (*p* < 0.05) (Figure 4). Moreover, with the progress of clinical classifications, the risk score of the high-risk groups also increased, which indicated that the nine-IRGP prognostic signature was closely related to clinical features (T, stage, and grade). However, no significant association was found between risk scores and other clinical features such as N, M, BMI, and age (Supplementary [Supplementary-material supplementary-material-1]).

**Figure 5:**
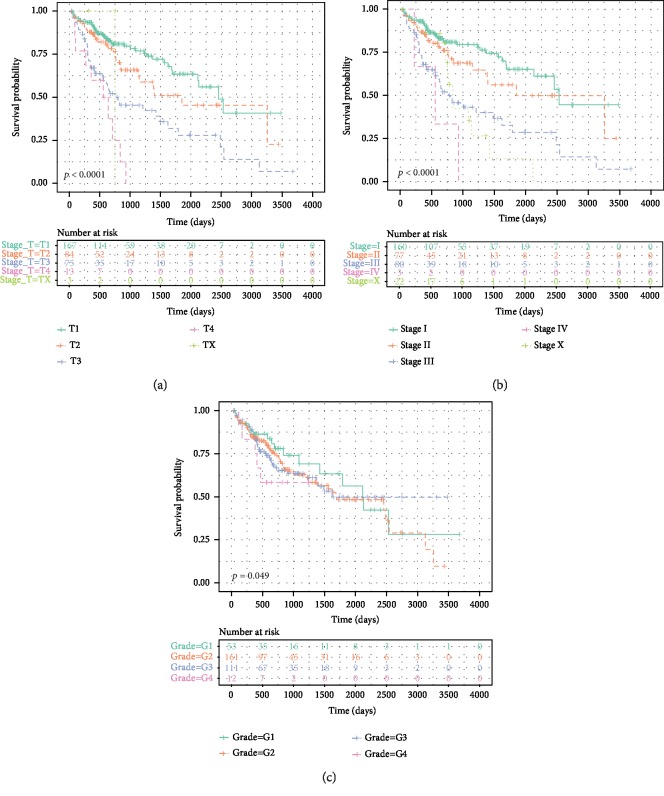
Kaplan–Meier survival analysis of the clinical classification (T, stage, and grade) based on the nine-IRGP signature. The survival analysis in (a) T, (b) stage, and (c) stage.

**Figure 6:**
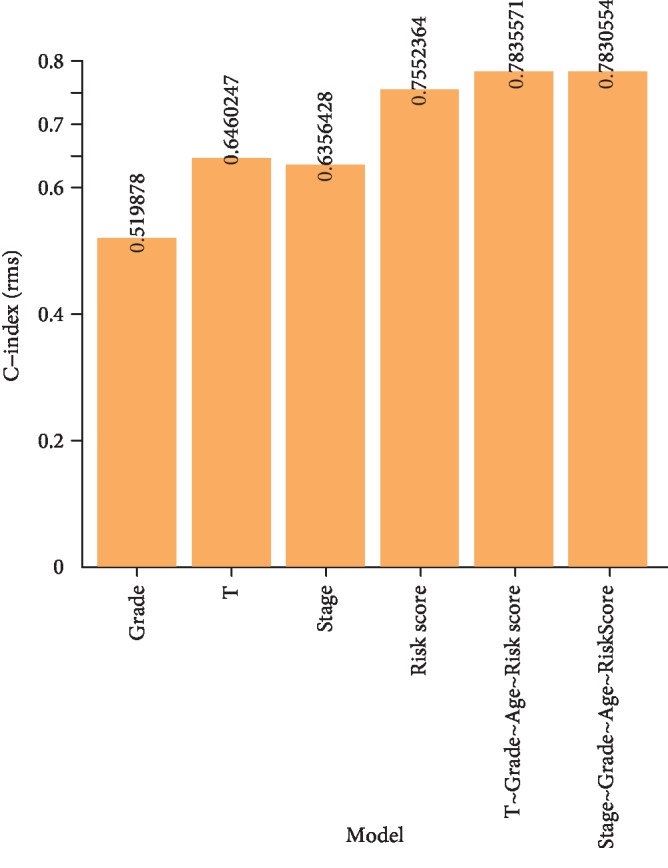
Comparison of C-index among multivariate prognostic modules, risk score, and clinical feature (T, stage, and grade).

Because T, stage, and grade were significantly associated with the prognosis of HCC patients based on the nine-IRGP signature, we investigated the OS for the T, stage, and grade. Consistent with clinical diagnosis, higher classification of T, stage, and grade correlated with worse prognosis of HCC samples (Figure 5). This further confirmed the accuracy of the prognostic assessment of the nine-IRGP signature. Furthermore, we constructed prognostic risk models of T, grade, and stage and compared these models with the nine-IRPG risk model. The nine-IRPG risk model achieved a higher C-index compared with T, grade, and stage risk modules. In addition, we established the multivariate prognostic modules, including T+grade+age+risk score and stage+grade+age+risk score. The C-index of the multivariate prognostic modules was not only higher than that of the nine-IRPG risk model but also was much higher than that of a single grade, T, or stage risk module (Figure 6). The result revealed that the nine-IRGP prognostic signature was more effective than the clinical feature in the prognosis of HCC patients. Hence, the nine-IRGP prognostic signature was verified as a robust complement to clinical features for the prognosis assessment of HCC patients.

We further investigated the association between nine IRGPs and other important HCC clinical features, such as the characteristic indexes (vascular invasion, bilirubin, and Child-Pugh stage) of the Barcelona Clinic Liver Cancer (BCLC) staging. The differences among groups were compared using the Kruskal–Wallis test. According to the results, the nine-IRPG risk score was associated with vascular invasion (*p* = 0.045), prothrombin time INR (*p* = 0.034), Child-Pugh (*p* = 0.050), and alpha-fetoprotein (AFP) (*p* = 0.008) (Supplementary [Supplementary-material supplementary-material-1]A–D). Notably, a higher nine-IRPG risk score was positively correlated to the vascular invasion, extension of prothrombin time INR, Child-Pugh B stage, and AFP > 400, which indicated that the nine-IRGP signature was a potential biomarker for predicting HCC progression. No significant association was observed between nine-IRPG risk score and total bilirubin (*p* = 0.37). However, the expression trend of the nine-IRPG risk score in AFP > 400 was higher than that of AFP ≤ 20 (Supplementary [Supplementary-material supplementary-material-1]E).

### 3.4. Functional Analysis of the Nine-IRGP Signature

**Figure 7:**
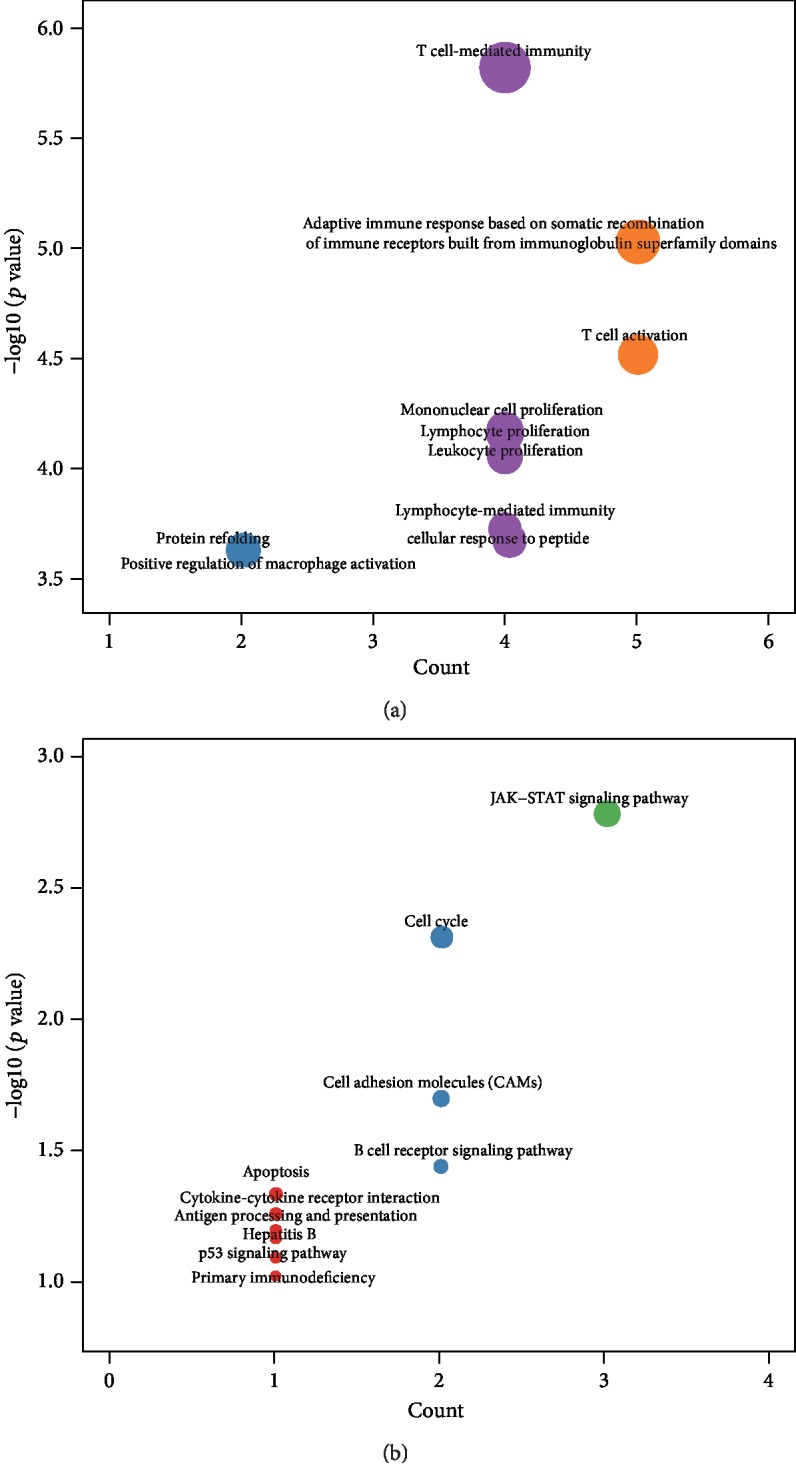
Functional analysis of the nine-IRGP signature. Biological function of the nine-IRGP signature for (a) GO and (b) KEGG analyses.

Given that the nine-IRGP signature was associated with the immune pathogenesis of HCC and it could separate HCC samples into the high and low-risk groups for prognostic prediction, we further analyzed the biological function of the nine-IRGP signature. Enrichment analysis of the GO terms revealed 302 items of biological processes, and most of the items were involved in the tumor immune mechanism such as cell-mediated immunity, immune response, and immune cell proliferation (Figure 7(a)). Furthermore, KEGG analysis identified 24 items of functional processes, including tumorigenesis signal pathway, cell cycle, apoptosis, and immune factors interaction. Our results revealed that the biological function of the nine-IRGP signature mainly related to the immune system's role in promoting or suppressing HCC development.

### 3.5. Forest and Nomogram Plots Analysis

**Figure 8:**
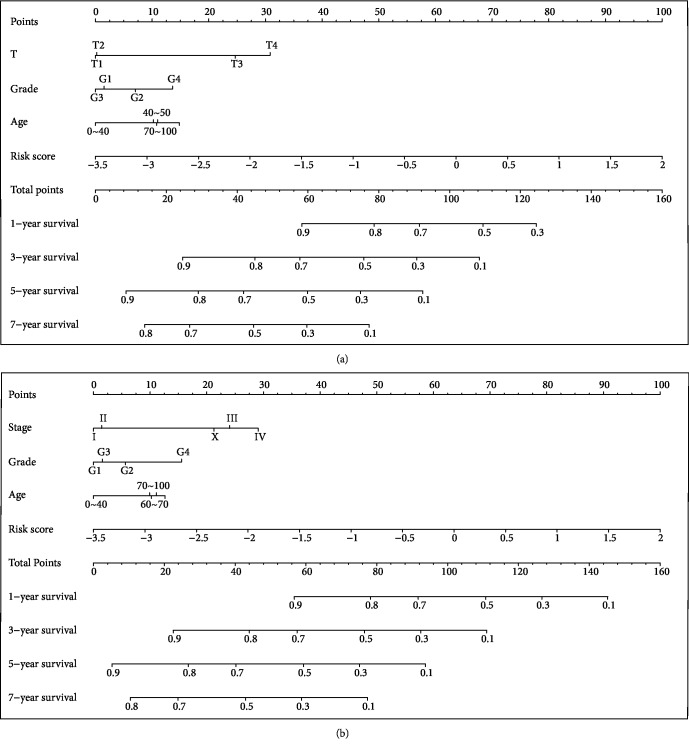
Prognostic value of nine IPGRs in HCC patients based on nomogram plots. (a) Clinical features (T, grade, and age) and risk score were analyzed to assess the survival time at 1, 3, 5, and 7 years for HCC patients. (b) Clinical features (stage, grade, and age) and risk score were analyzed to assess the survival time at 1, 3, 5, and 7 years for HCC patients.

**Figure 9:**
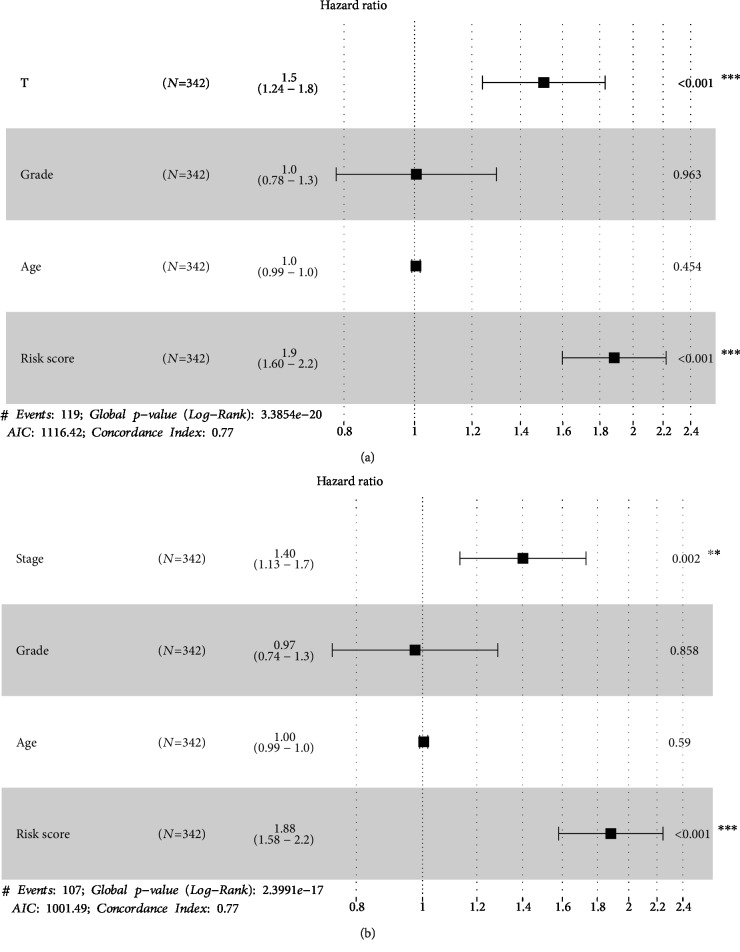
Prognostic value of the nine IPGRs in HCC patients based on forest plots. (a) Clinical features (T, grade, and age) and risk score were analyzed to assess the hazard ratio for HCC patients. (b) Clinical features (stage, grade, and age) and risk score were analyzed to assess the hazard ratio for HCC patients.

For systematic verification of the prognostic value of the nine IPGR signature in HCC patients, we constructed nomogram plots by combining risk score and independent clinical risk factors (T, grade, stage, and age). In the nomogram plot, the length of the line indicated the degree of influence of different factors on the outcome, as well as the effect of different values of factors on the outcome. The nomogram plots showed that the risk score based on nine IRGPs had the longest line indicating it had the greatest influence on the prediction of survival rate. The nomogram plots also revealed that the nine IRGPs contributed the highest number of risk points (from 0 to 100) compared to other clinical features, which was consistent with the results of multivariate prognostic modules analyses (Figure 8). Moreover, forest plots were constructed to display the statistical summary results of risk score and different clinical factors (T, grade, stage, and age). In the forest map, several line segments parallel to the *x*-axis represented the effect of factors and its 95% confidence interval. The hazard ratio (HR) value of risk score based on the nine IRGPs was the highest among the factors (Figure 9). In T+grade+age+risk score module and stage+grade+age+risk score module, the HR value of risk score was about 1.9 and the *p* < 0.001. This result was also consistent with the analysis of multivariate prognostic modules which showed that the nine-IRGP signature was the most effective signature for prognostic assessment of HCC patients when compared with other clinical features. These outcomes further confirmed that the nine-IRGP signature could be an effective biomarker for estimating the prognosis of HCC patients.

## 4. Discussion

Patients usually lack obvious clinical symptoms during the early stages of HCC, and even after detection and treatment, the patients remain at risk of tumor recurrence or progression [21]. Although clinical markers such as alpha-fetoprotein (AFP), glutamyl transpeptidase (*γ*-GT), and lactic dehydrogenase (LDH) have been used in the early diagnosis and therapeutic monitoring of HCC patient, these factors are nonspecific. Also, these clinical markers are not elevated in most of the patients at initial stages of HCC and do not reflect the severity of the disease [22]. Thus, there is a need to find new early diagnostic markers of HCC. Herein, we constructed a nine-IRGP signature for HCC patients and validated its accuracy and effectiveness in an independent data set through multidimensional bioinformatics methods. The nine-IRGP signature was able to group HCC patients into the high and low-risk groups based on OS and was also superior to other clinical risk factors (T, grade, stage, and age) in the prognostic assessment. It is noteworthy that multidimensional bioinformatics methods are used in the study of large tumor samples for biomarker mining. Nault et al. conducted multiomics to comprehensively analyze the genomic profiling of HCC combined with tumor stages, clinical features, and survival. They reported molecular prognostic 5-gene score, which could show different distributions according to the stage of the disease, type of treatment, prognostic score, and pathologic types. Also, they highlighted the multiomics for genomic analysis on HCC to reveal the mechanisms of HCC and helped to identify biomarkers for response to targeted therapies [23]. Therefore, it was feasible for us to apply multidimensional bioinformatics methods in the mining of the biomarkers of HCC based on several data sets. We combined gene expression profiles from multiple data sets for gene pairwise analysis to identify reliable biomarkers for prognosis of HCC. In doing so, the bias caused by gene normalization was eliminated [24]. There was a significant reduction in the difference between the GEO and TCGA platforms after gene pairwise conversion of IRGs to IRGPs. The validation of the nine-IRGP signature was comprehensive, including univariate analysis, multivariate analysis, forest plot, and nomogram plots. The multidimensional bioinformatics methods are more rigorous in verifying the reliability of biomarkers [25]. Moreover, we combined the nine IRGPs with the clinical/pathologic features and used them to establish risk modules for comparative analysis. According to the results, the nine-IRGP signature was a robust complement to several features (T, stage, and grade) for HCC prognostic prediction, and significant prognostic performance was achieved by the combination of the nine IRGPs and clinical/pathologic features. These results confirmed that the nine-IRGP signature was accurate, considering that the data set resource was adequate, the analytical methods were comprehensive, and the validation was combined with clinical features. Furthermore, we found that the nine-IRGP signature could stratify HCC into the high- and low-risk groups and revealed that the low-risk group had a better prognosis than the high-risk group. This observation was consistent with a previous study by Sia et al. However, Sia et al. focused on the genotyping of HCC, as well as the immune microenvironment and oncogenic signaling pathways of HCC, and therefore, their study provided a more reliable HCC classification than the present study [17]. According to Calderaro et al., high immune response with more infiltrated immune cells results in frequent gene mutation. On the contrary, exhausted immune response with less infiltration of immune cells represents a nonproliferative tumor characterized by chromosomal stability and maintenance of hepatocytic marker expression [18]. Kurebayashi et al. conducted comprehensive analyses of immune cells through multiplex immunohistochemistry and classified the immune microenvironment of HCC into three distinct immune subtypes (immune-high, immune-mid, and immune-low). Contrary to our results, they found that the immune-high subtype was characterized by increased B/plasma cell and T cell infiltration was associated with good prognosis [26]. We hypothesized that the composition of immune cells plays a vital role in the pathogenesis of tumors. Immune activation by B cell, T cell, natural cell, and dendritic cell contributes to the tumor suppression [27]. Therefore, further study of this immune classifier, including immune microenvironment, immune-related gene mutation, and immune infiltrated cell component, should be conducted to verify the nine-IRGP signature as validated biomarker for HCC prognosis.

Studies have showed that biomarkers based on tumor immunity can be used for diagnosis, treatment, and prognosis of cancer patients [28]. The immune genes identified in this study were associated with several immune functions, such as cellular response to cytokine, cell mediation of immunity, immune signaling pathway, and immune response to tumor cell. Similarly, the immune-related genes of the nine-IRGP signature played a significant role in tumor immunological mechanism. Palmer et al. showed that CISH could prevent the recognition of tumor cells by weakening the biological function of the T cell receptor (TCR) signal in CD8 T cells [29]. TYRO3 was found to be involved in the biological process of immune regulation and promoted tumor cell proliferation, metastasis, and chemotherapy resistance. Moreover, higher expression levels of TYRO3 were negatively correlated with low survival rate in HCC patients [30]. SOCS2 proteins are important negative regulators of cytokine signal transduction, and their inhibition may be an effective therapeutic strategy for cancer treatment [31]. Other immune-related genes of the nine-IRGP signature, including BTN3A3 [32], RORC [33], and AGER [34], participate in promoting tumorigenesis, decreasing efficacy of immunotherapy, and preventing tumor immune killing through a variety of immunological mechanisms. In the present study, we observed that the HCC patients in the high-risk group with high expression of nine IRGPs had poor prognosis, indicating that nine IRGPs promoted tumorigenesis. And this was consistent with the finding of previous studies. Functional analysis of nine IRGPs revealed that they participated in many tumor immune mechanisms, including cell-mediated immunity, immune response, and immune cell proliferation, as well as tumorigenesis signal pathway, cell cycle, apoptosis, and immune factors interaction. T cell-mediated immunity plays a crucial role in inhibiting tumor proliferation. Cytotoxicity T lymphocytes (CTL) provide effective antitumor immunity in vivo, especially in immunosuppressive patients with more viral infection-related tumors, such as HCC [35]. Contrary to the findings, immune cell proliferation contributes to the killing of tumor cells; recent studies have found that tumor cells can recruit neutrophils to drive tumorigenesis and metastasis [36]. Also, immune-related tumorigenesis signal pathways, such as TGF-*β* [37], Hippo [38], and cGAS-STING [39], can promote inflammatory reactions, thereby enhancing tumorigenesis. The nine IRGPs identified in this study were involved in a wide range of immune processes, which may explain why the nine-IRGP signature could be a robust biomarker for predicting the prognosis of HCC.

## 5. Conclusion

There are several limitations to this study. First, our study was based on the typing of immune genes, but we ignored the role of immune cell infiltration in the tumor immune microenvironment. Many studies have found that the classification of HCC based on immune cell infiltration was closely related to clinical characteristics. Second, the HCC cohort in our study did not contain the comparison between the cases with immune-checkpoint inhibitor therapy and normal therapy. Therefore, we could not analyze the effect of immune-related genes biomarker in the immunotherapy. Third, the associated analysis between immune-related genes and clinical features was not enough; thus, we should conduct additional clinical features, such as Barcelona Clinic Liver Cancer (BCLC) stage and Child-Pugh grading. Given this, the clinical application of the nine-IRGP signature is not very clear and needs further validation. In the current study, we constructed a nine-IRGP signature based on multigene expression data set for the prognostic prediction of HCC patients. These results suggest that the nine-IRGP signature may be a potential biomarker for the prognosis of HCC patients.

## Figures and Tables

**Table 1:** Information about the nine-IRGP signature.

Gene name	Coefficient
CISH_vs_PIAS3	-0.293734419
CISH_vs_HSPA14	-0.012715098
SOCS2_vs_TYRO3	-0.174114652
ACAP1_vs_CD180	-0.294342403
MAP3K3_vs_BTN3A3	0.111743851
TRIB3_vs_RORC	0.023271926
AGER_vs_TYRO3	-0.037584122
SDC4_vs_HSPD1	-0.039802843
PLAUR_vs_CD8A	0.006054132

## Data Availability

The data used to support the findings of this study are available in the public databases.

## Supplementary Materials

Supplementary 1 Supplementary Figure 1: consistency evaluation of data sets on different platforms. Cluster analysis of (A) IRGs and (B) IRGPs from TCGA, GSE14520, and GSE76427 database sets. Differences between the three database sets based on (C) IRG and (D) IRGP platforms.

Supplementary 2 Supplementary Figure 2: association between clinical features (T, stage, and grade) and risk score based on the nine-IRGP signature. Distribution of risk scores in (A) N, (B) M, (C) age, and (D) BMI for the TCGA data set.

Supplementary 3 Supplementary Figure 3: association between characteristic indexes of the Barcelona Clinic Liver Cancer (BCLC) staging and nine-IRPG risk score. Distribution of the nine-IRPG risk score in groups with different statuses of vascular invasion (A), prothrombin time INR (B), total bilirubin (C), Child-Pugh (D), and alpha-fetoprotein (AFP) (E).

Supplementary 4 Supplementary Table 1: three database sets of clinical information after preprocessing.

Supplementary 5 Supplementary Table 2: clinical information statistics of training and testing sets.
