# Supplementary material for: Identification and Validation of Immune-Related Gene Prognostic Signature for Hepatocellular Carcinoma
Source: J Immunol Res. 2020 Mar 7;2020:5494858. doi: 10.1155/2020/5494858 (PMC7081044; doi:10.1155/2020/5494858)
Supplement: Supplementary 4 — Supplementary Table 1: three database sets of clinical information after preprocessing. [file 5494858.f4.docx]

Table 1. Three database sets of clinical information after preprocessing.

|  | **TCGA** | **GSE14520** | **GSE76427** |
| --- | --- | --- | --- |
| **Event** |  |  |  |
| Alive | 219 | 136 | 75 |
| Dead | 123 | 84 | 20 |
| **T** |  |  |  |
| T1 | 167 |  |  |
| T2 | 84 |  |  |
| T3 | 75 |  |  |
| T4 | 13 |  |  |
| TX | 3 |  |  |
| **N** |  |  |  |
| N0 | 238 | 56 |  |
| N1 | 3 | 155 |  |
| NX | 101 | 9 |  |
| M |  |  |  |
| M0 | 244 | 80 |  |
| M1 | 3 | 139 |  |
| MX | 95 | 1 |  |
| **Stage** |  |  |  |
| I | 160 | 93 |  |
| II | 77 | 77 |  |
| III | 80 | 48 |  |
| IV | 3 | 2 |  |
| X | 22 |  |  |
| **Grade** |  |  |  |
| G1 | 53 |  |  |
| G2 | 161 |  |  |
| G3 | 111 |  |  |
| G4 | 12 |  |  |
| **Age** |  |  |  |
| 0~40 | 29 | 30 | 2 |
| 40~50 | 36 | 66 | 9 |
| 50~60 | 91 | 82 | 30 |
| 60~70 | 112 | 31 | 24 |
| 70~100 | 74 | 11 | 30 |
| **BMI** |  |  |  |
| 0~18.5 | 45 |  |  |
| 18.5~25 | 144 |  |  |
| 25~30 | 89 |  |  |
| 30~100 | 64 |  |  |
