# Supplementary material for: Identification and Validation of Immune-Related Gene Prognostic Signature for Hepatocellular Carcinoma
Source: J Immunol Res. 2020 Mar 7;2020:5494858. doi: 10.1155/2020/5494858 (PMC7081044; doi:10.1155/2020/5494858)
Supplement: Supplementary 5 — Supplementary Table 2: clinical information statistics of training and testing sets. [file 5494858.f5.docx]

Table 2. Clinical information statistics of training and testing sets.

| **Clinical Features** | **Training Set** | **Testing Set** |
| --- | --- | --- |
| **Event** |  |  |
| Alive | 107 | 112 |
| Dead | 63 | 60 |
| **T** |  |  |
| T1 | 77 | 90 |
| T2 | 43 | 41 |
| T3 | 39 | 36 |
| T4 | 9 | 4 |
| TX | 2 | 1 |
| **N** |  |  |
| N0 | 116 | 122 |
| N1 | 1 | 2 |
| NX | 53 | 48 |
| **M** |  |  |
| M0 | 125 | 119 |
| M1 | 2 | 1 |
| MX | 43 | 52 |
| **Stage** |  |  |
| I | 75 | 85 |
| II | 40 | 37 |
| III | 42 | 38 |
| IV | 2 | 1 |
| X | 11 | 11 |
| **Grade** |  |  |
| G1 | 31 | 22 |
| G2 | 76 | 85 |
| G3 | 53 | 58 |
| G4 | 7 | 5 |
| **Age** |  |  |
| 0~40 | 12 | 17 |
| 40~50 | 19 | 17 |
| 50~60 | 46 | 45 |
| 60~70 | 60 | 52 |
| 70~100 | 33 | 41 |
| **BMI** |  |  |
| 0~18.5 | 22 | 23 |
| 18.5~25 | 72 | 72 |
| 25~30 | 39 | 50 |
| 30~100 | 37 | 27 |
